# Supplementary material for: Activated endothelial cells induce a distinct type of astrocytic reactivity
Source: Commun Biol. 2022 Mar 29;5:282. doi: 10.1038/s42003-022-03237-8 (PMC8964703; doi:10.1038/s42003-022-03237-8)
Supplement: Supplementary file 2 — Supplementary Information [file 42003_2022_3237_MOESM2_ESM.pdf]

Supplementary Information

Activated Endothelial Cells Induce a Distinct Type of Astrocytic Reactivity

Xavier Taylor[1,2], Pablo Cisternas[1,2], Nur Jury[1,2], Pablo Martinez[1,2], Xiaoqing Huang[3], Yanwen You[1,2], Javier Redding-Ochoa[4], Ruben Vidal[1,5], Jie Zhang[3,6], Juan Troncoso[4], Cristian A. Lasagna-Reeves[1,2,6*]

Supplementary-Figure 1.

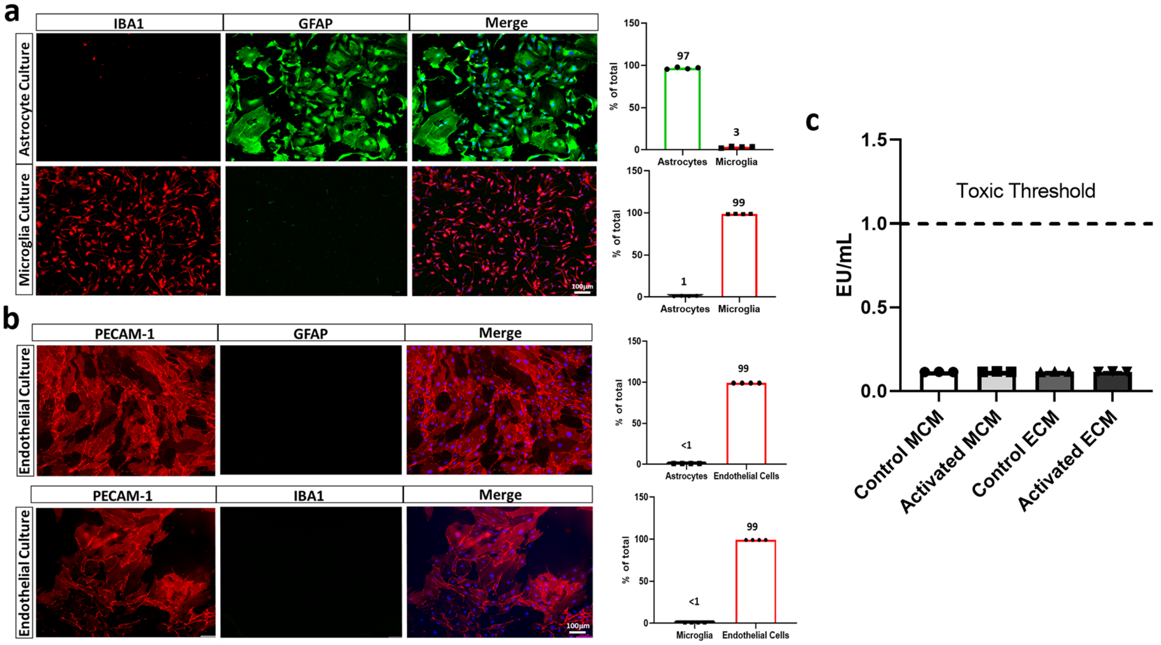

Supplementary-Figure 1. Cell culture purity analysis. a) Double immunofluorescence of astrocytes (GFAP, green) and microglia (IBA1, red) in primary astrocyte (top image) and microglia (bottom image) cultures. **b)** Double immunofluorescence of brain microvascular endothelial cells (PECAM-1, red) and astrocytes or microglia (GFAP or IBA1, green) in primary endothelial cultures. c) The level of endotoxin in conditioned media was measured by using the Pierce LAL Chromogenic Endotoxin Quantification Kit (Thermo Fisher Scientific Inc, Rockford, IL,USA). The data represents the median ± IQR of *n*=≥3. Scale bar 100 µm.

Supplementary Figure 2.

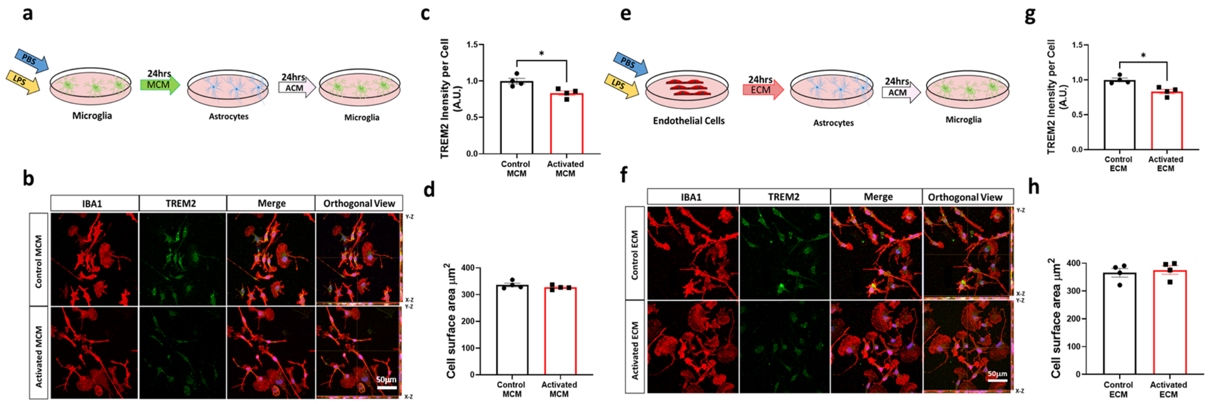

Supplementary Figure 2. ACM from activated MCM and ECM treatments decreases TREM2 protein expression in mouse primary microglia cultures. **a)** Schematic diagram showing treatment of microglia with astrocytic conditioned medium (ACM) from previous treatment with control or activated microglial conditioned medium (MCM). **b)** Double immunofluorescence images of microglia (IBA1, red) and TREM2 (green) of primary WT mouse microglia culture treated with ACM. The right panel shows orthogonal images. **c)** Intensity quantification of TREM2 per cell shows a decrease in TREM2 protein expression in microglia incubated with ACM from control or activated MCM treatment **d)** Quantification of $IBA1^+$ cell surface area shows no change in cell surface area between ACM from control or activated MCM treatment. **e)** Schematic diagram showing treatment of microglia with ACM from previous treatment with control or activated endothelial conditioned media (ECM). **f)** Double immunofluorescence images of microglia (IBA1, red) and TREM2 (green) of primary WT mouse microglia culture treated with ACM from control ECM treatment or ACM from Activated ECM treatment. The right panel shows orthogonal images. **g)** Intensity quantification of TREM2 per cell shows a decrease in TREM2 proteins in microglia incubated with ACM from control or activated ECM **h)** Quantification of $IBA1^+$ cell surface area shows no change in cell surface area between ACM from control and activated ECM treatment. For quantifications, error bars represented median ± IQR where eight to ten images were used for each experiment with $n = 4$ independent cultures and 20 cells per image, where $*p < 0.05$ by Mann-Whitney test. Scale bar 50 µm.

Supplementary Figure 3.

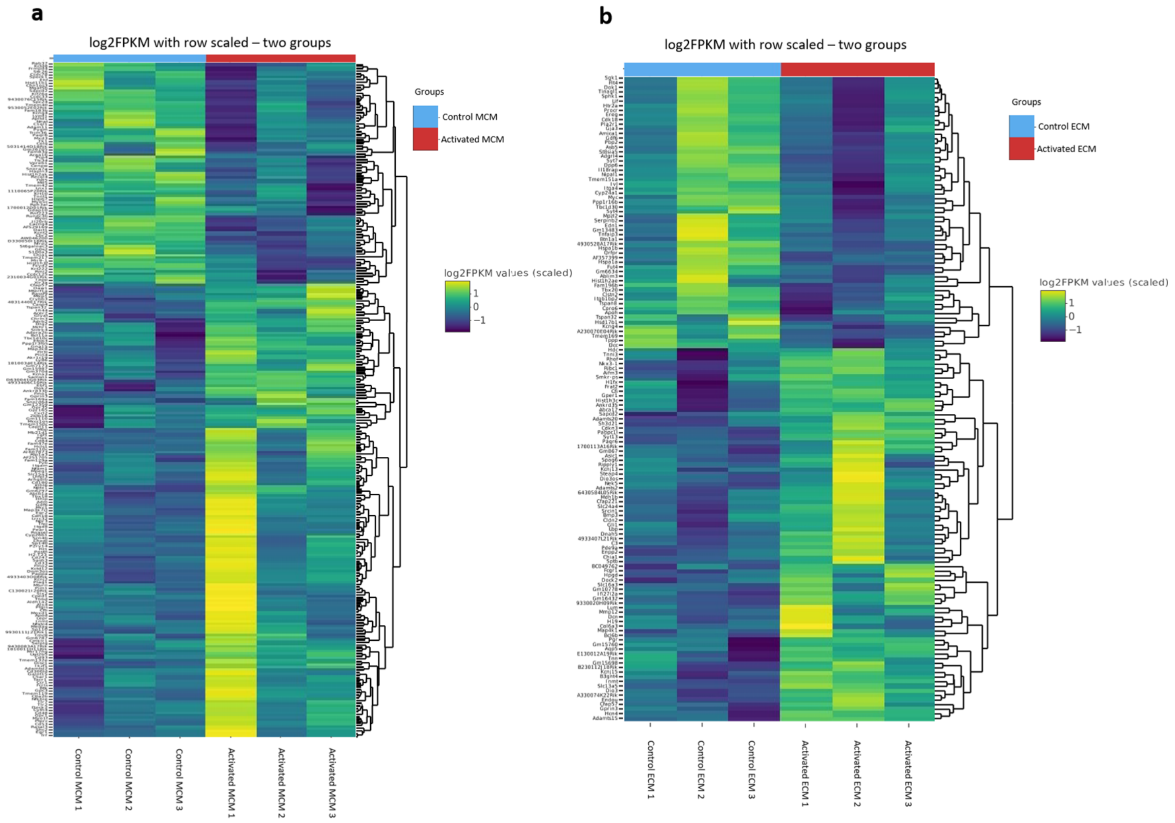

Supplementary Figure 3. Microglial and endothelial induced astrocytic transcriptome. a) RNA-seq generated heatmap of gene expression profiles of astrocytes activated by microglia conditioned media. **b)** RNA-seq generated heatmap of gene expression profiles of astrocytes activated by endothelial conditioned media (yellow = upregulation, purple = downregulation).

Supplementary Figure 4.

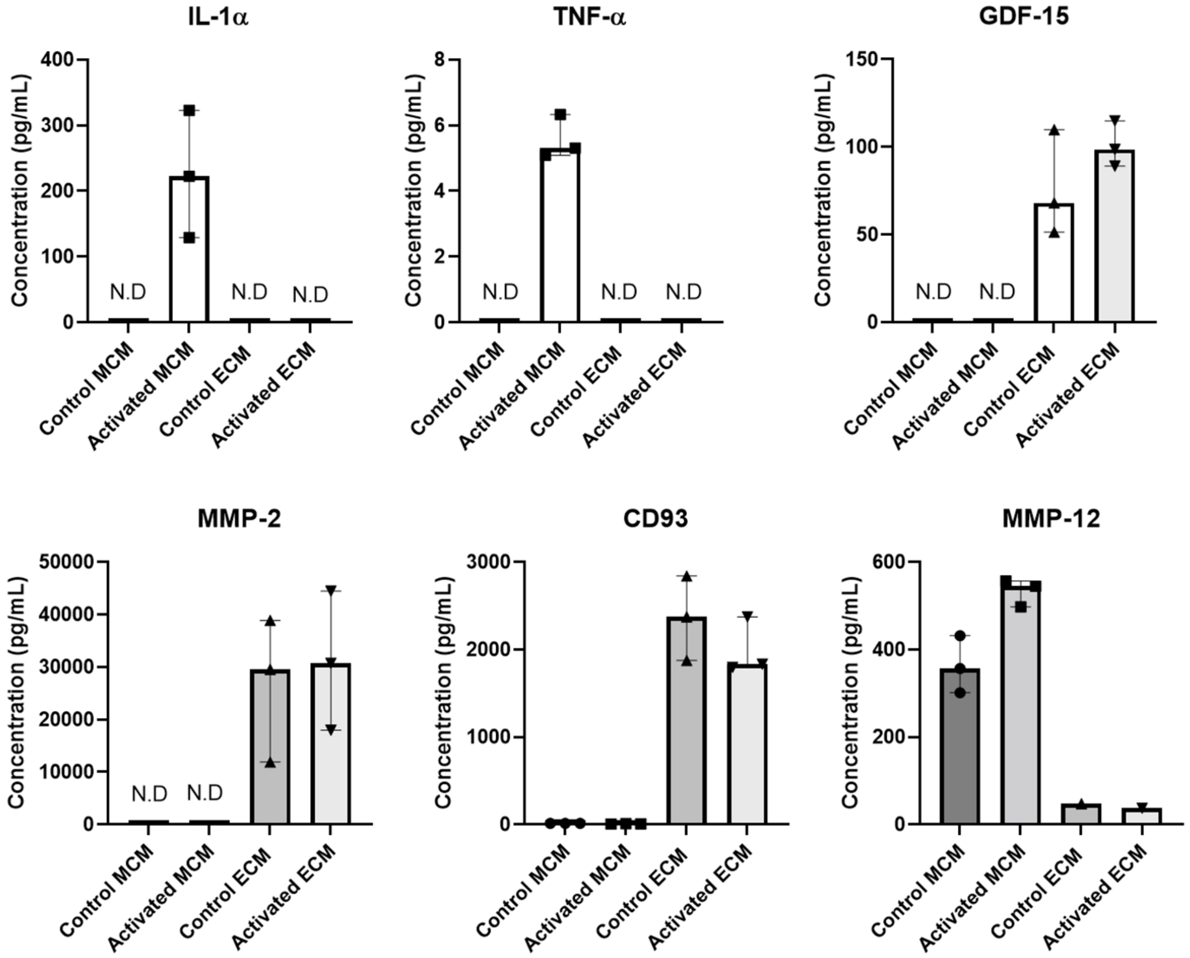

Supplementary Figure 4. Luminex-based multiplex assay. Concentration (pg/mL) of IL-1α, TNF-α, GDF-15, MMP-2, CD93 and MMP12 in cell culture media in control vs activated microglia and endothelial conditioned media (N.D = not detected). Results are shown as the median ± IQR of *n*=3.

Supplementary-Figure 5.

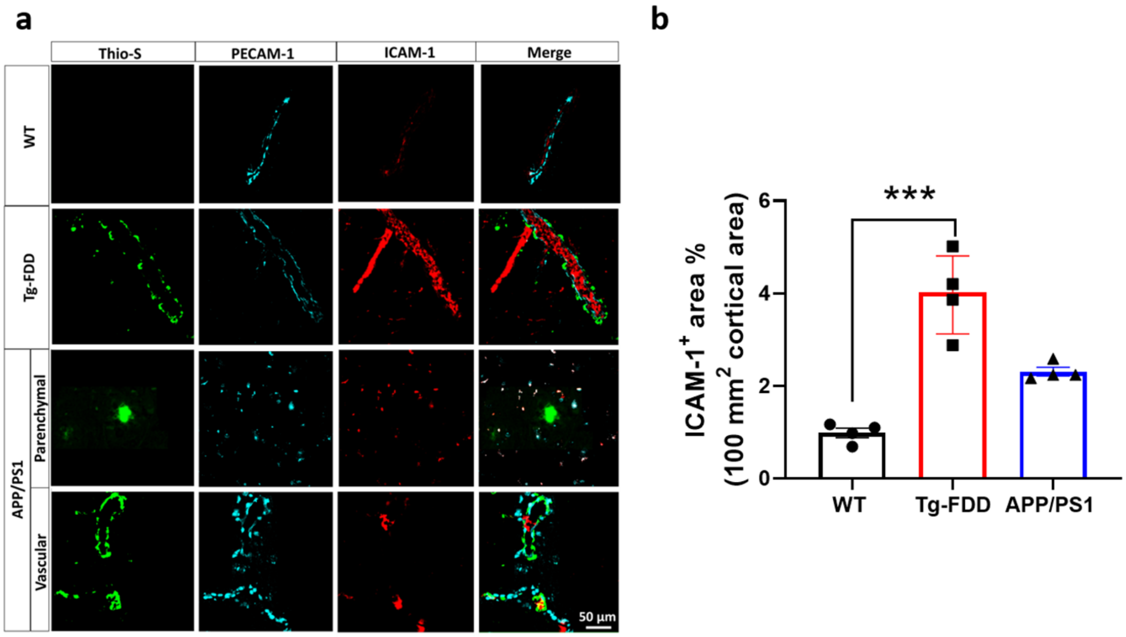

Supplementary-Figure 5. Damaged endothelial cells are associated with CAA. a) Triple immunofluorescence for amyloid (Thio-S, green), endothelial cells (PECAM-1, Cyan) and activated endothelium marker (ICAM-1, red) in Tg-FDD, APP/PS1 and WT. Thio-S, PECAM-1 and ICAM-1 immunoreactivity overlay (Merge). **b)** Quantification of ICAM-1^{+} area (%) show ICAM-1^{+} staining is associated with vascular amyloid but not parenchymal amyloid deposits. The results are shown as the median ± IQR of $n = 4$. Asterisks indicate significant differences, where *** $p < 0.001$ by Kurskal-Wallis test. Scale bar 50 µm.

Supplementary Figure 6.

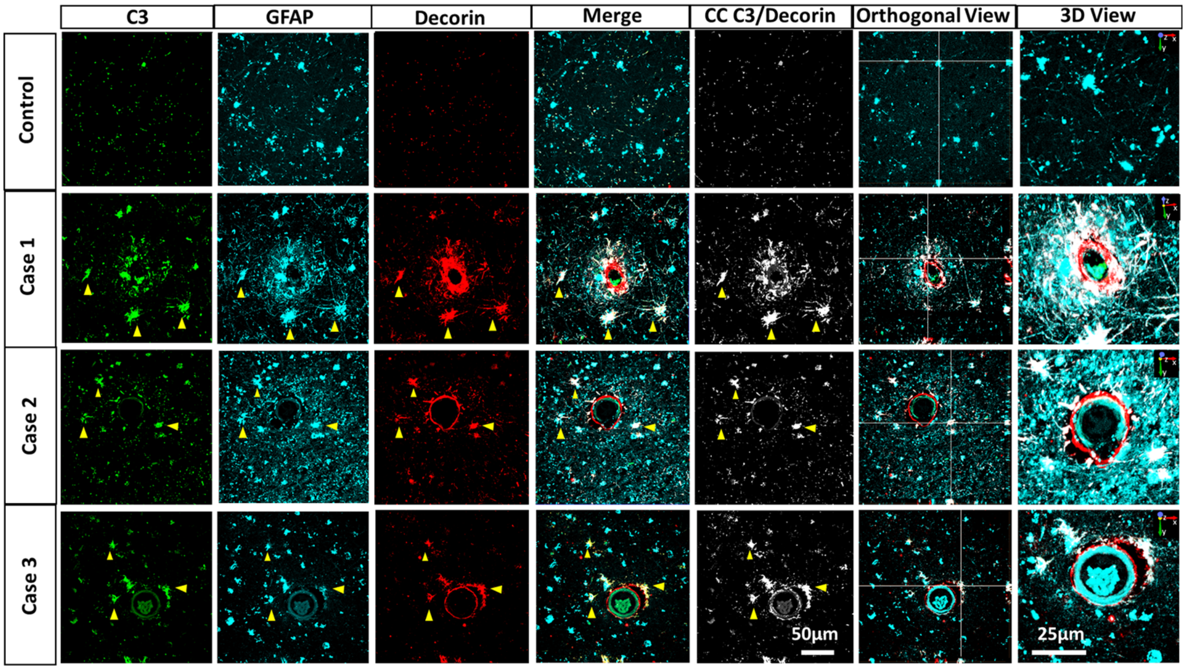

Supplementary Figure 6. Decorin^{+} C3^{+} astrocytes are present in AD/CAA patients. Triple immunofluorescence of complement component 3 (C3, green), astrocytes (GFAP, cyan), and Decorin (red) in AD/CAA and healthy control patients show astrocytes colocalizing with Decorin^{+} C3^{+} staining. C3, GFAP and Decorin immunoreactivity overlay (Merge). Yellow arrows indicated C3^{+}, GFAP^{+}, Decorin^{+} positive cells. Colocalization analysis (CC) was performed to determine pixel intensity correlation between Decorin and GFAP. White pixels indicate colocalization between C3 and Decorin signal. The right panel shows orthogonal images and reconstructed three-dimensional views. Scale bar 25 & 50 µm, respectivley.

Supplementary Figure 7.

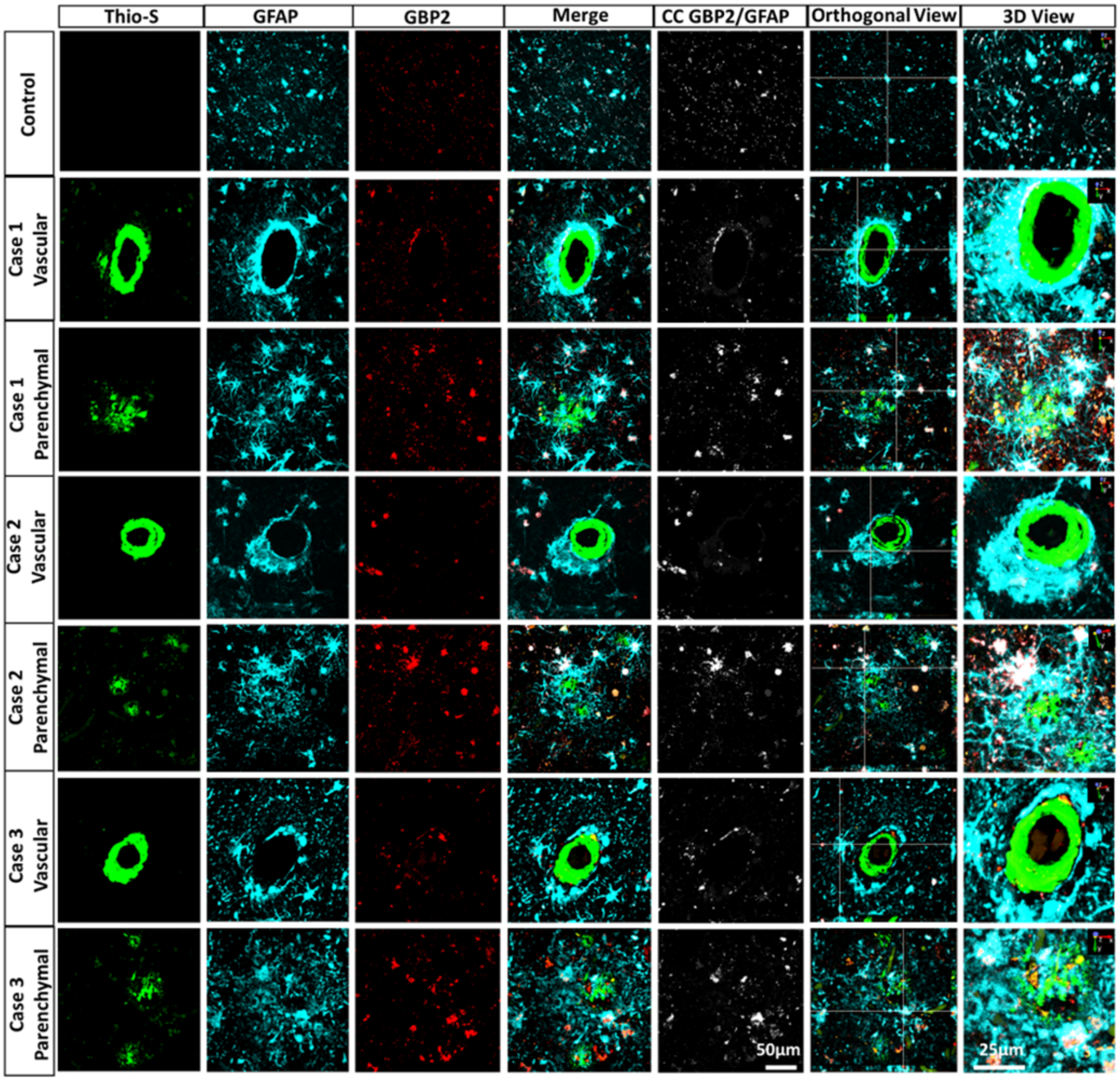

Supplementary Figure 7. GBP2⁺ astrocytes are associated with parenchymal amyloid plaques but not with vascular amyloid in AD/CAA patients. Triple immunofluorescence for amyloid (Thio-S, green), astrocytes (GFAP, Cyan) and GBP2 (red) in AD/CAA and healthy control patients show GBP2⁺ staining is associated with parenchymal amyloid deposits but not with vascular amyloid deposits. Thio-S, GFAP and GBP2 immunoreactivity overlay (Merge). Colocalization analysis (CC) was performed to determine pixel intensity correlation between GBP2 and GFAP. White pixels indicate colocalization between GBP2 and GFAP signal. The right panel shows orthogonal images and reconstructed three-dimensional views. Scale bar 25 & 50 µm, respectively.

Supplementary Table 1.

CASE #	FDX	THAL	CERAD	BRAAK	AGE	SEX	RACE	PMD	Brain Region
Control	Control	NA	0	2	85	F	W	27	OCC
1	AD, CEREBRAL AMYLOID ANGIOPATHY	NA	C	5	75	M	W	13	OCC
2	MIXED DEMENTIA AD+VASCULAR, AD, VASCULAR DEMENTIA	NA	C	5	78	M	B	5	OCC
3	AD, CVD (NC)	5	C	6	79	M	W	15	OCC
4	AD, CAA (MINIMAL)	NA	C	6	73	F	W	6	OCC
5	AD PROBABLE, CAA (MINIMAL)	NA	B	4	87	F	W	33	OCC
6	CAA, Asymptommatic AD, lobar hemorhage	5	C	6	84	F	W	27	OCC

Supplementary Table 1. Human AD and CAA cases. FDX, final diagnosis; AD, Alzheimer's disease; CAA, Cerebral Amyloid Angiopathy, Control, healthy individual without observed pathology; CVD, cardiovascular disease; Thal 1-5; Cerad A-C; Braak 1-5; F, female; M, male; W, White; B, Black; PMD, Post Mortem Delay, OCC, Occipital cortex.
