# Supplementary material for: Activated endothelial cells induce a distinct type of astrocytic reactivity
Source: Commun Biol. 2022 Mar 29;5:282. doi: 10.1038/s42003-022-03237-8 (PMC8964703; doi:10.1038/s42003-022-03237-8)
Supplement: Supplementary file 3 — Description of Additional Supplementary Files [file 42003_2022_3237_MOESM3_ESM.pdf]

Description of Additional Supplementary Files

File name: Supplementary Data 1
Description: NanoString gene set enrichment analysis**.**

File name: Supplementary Data 2
Description: RNA Sequencing Analysis.

File name: Supplementary Data 3
Description: Source data underlying the graphs and charts in the main manuscript.
