# Supplementary material for: Activated endothelial cells induce a distinct type of astrocytic reactivity
Source: Commun Biol. 2022 Mar 29;5:282. doi: 10.1038/s42003-022-03237-8 (PMC8964703; doi:10.1038/s42003-022-03237-8)
Supplement: Supplementary file 7 — Reporting Summary [file 42003_2022_3237_MOESM7_ESM.pdf]

nature portfolio

Corresponding author(s): Cristian-Lasagna Reeves
Last updated by author(s): Feb 1, 2022

Reporting Summary

Nature Portfolio wishes to improve the reproducibility of the work that we publish. This form provides structure for consistency and transparency in reporting. For further information on Nature Portfolio policies, see our Editorial Policies and the Editorial Policy Checklist.

Statistics

For all statistical analyses, confirm that the following items are present in the figure legend, table legend, main text, or Methods section.

n/a	Confirmed	
☐	☒	The exact sample size (*n*) for each experimental group/condition, given as a discrete number and unit of measurement
☐	☒	A statement on whether measurements were taken from distinct samples or whether the same sample was measured repeatedly
☐	☒	The statistical test(s) used AND whether they are one- or two-sided<br>*Only common tests should be described solely by name; describe more complex techniques in the Methods section.*
☒	☐	A description of all covariates tested
☐	☒	A description of any assumptions or corrections, such as tests of normality and adjustment for multiple comparisons
☐	☒	A full description of the statistical parameters including central tendency (e.g. means) or other basic estimates (e.g. regression coefficient) AND variation (e.g. standard deviation) or associated estimates of uncertainty (e.g. confidence intervals)
☐	☒	For null hypothesis testing, the test statistic (e.g. *F*, *t*, *r*) with confidence intervals, effect sizes, degrees of freedom and *P* value noted<br>*Give P values as exact values whenever suitable.*
☒	☐	For Bayesian analysis, information on the choice of priors and Markov chain Monte Carlo settings
☒	☐	For hierarchical and complex designs, identification of the appropriate level for tests and full reporting of outcomes
☒	☐	Estimates of effect sizes (e.g. Cohen's *d*, Pearson's *r*), indicating how they were calculated

Our web collection on statistics for biologists contains articles on many of the points above.

Software and code

Policy information about availability of computer code

Data collection	RNA seq, Nanostring, Epifluorescent and Confocal Imaging.
Data analysis	RNA Sequencing quality FastQC/MultiQC 0.11.5, Alignment STAR 2.5 (Dobin et al. 2012), Mapping quality NGSUtils,bamutils 0.5.9 (Breese and Liu 2013), Read counting subread/featureCounts 1.5.1 (Liao, Smyth, and Shi 2013), Expression analysis edgeR 3.28.1 (Robinson, Mccarthy, and Smyth 2010). NanoString nSolver Analysis Software 4.0.70. Imaging data were processed and analyzed using Image J (NIH, v1.53i). Data was statistically analyzed using GraphPad Prism (v9.0).

For manuscripts utilizing custom algorithms or software that are central to the research but not yet described in published literature, software must be made available to editors and reviewers. We strongly encourage code deposition in a community repository (e.g. GitHub). See the Nature Portfolio guidelines for submitting code & software for further information.

Data

Policy information about availability of data

All manuscripts must include a data availability statement. This statement should provide the following information, where applicable:

- Accession codes, unique identifiers, or web links for publicly available datasets
- A description of any restrictions on data availability
- For clinical datasets or third party data, please ensure that the statement adheres to our policy

The accession numbers for the RNA-seq and NanoString data reported in this paper are Gene Expression Omnibus (GEO): GSE175485 & GSE180106, respectively.

Field-specific reporting

Please select the one below that is the best fit for your research. If you are not sure, read the appropriate sections before making your selection.

☒ Life sciences ☐ Behavioural & social sciences ☐ Ecological, evolutionary & environmental sciences

For a reference copy of the document with all sections, see nature.com/documents/nr-reporting-summary-flat.pdf

Life sciences study design

All studies must disclose on these points even when the disclosure is negative.

Sample size	No statistical methods were performed to predetermine sample size. Sample sizes were chosen based on literature to determine transcriptional and biochemical changes. The sample size (n) of each experiment is provided in the figure legends.
Data exclusions	No data were excluded from any analysis performed.
Replication	All experiments were repeated at least three times, and the results are reproducible. Imaging and transcriptional experiments were performed using different cohorts of animals. The results are consistent and robust.
Randomization	Animals were randomly selected for the experimental and control groups with matched age and sex, whenever possible. Animal sections used for imaging were also selected randomly.
Blinding	All the experiment and analysis were performed blindly.

Reporting for specific materials, systems and methods

We require information from authors about some types of materials, experimental systems and methods used in many studies. Here, indicate whether each material, system or method listed is relevant to your study. If you are not sure if a list item applies to your research, read the appropriate section before selecting a response.

Materials & experimental systems

n/a	Involved in the study	
☐	☒	Antibodies
☒	☐	Eukaryotic cell lines
☒	☐	Palaeontology and archaeology
☐	☒	Animals and other organisms
☒	☐	Human research participants
☒	☐	Clinical data
☒	☐	Dual use research of concern

Methods

n/a	Involved in the study	
☒	☐	ChIP-seq
☒	☐	Flow cytometry
☒	☐	MRI-based neuroimaging

Antibodies

Antibodies used	Primary Antibodies: anti-GFAP (G3893, Sigma-Aldrich), anti-IBA (1019-19741, Wako), anti-CD 31 (PECAM-1) (1:100, PA5-16301, ThermoFisher), anti-ICAM-1 (MA5407, ThermoFisher) anti-C3 (PA5-21349, ThermoFisher), anti-Synapsin-1(ab64581, Abcam), anti-PSD95 (ab2723, Abcam), anti-Decorin (14667-1-AP, ThermoFisher), anti-GBP2 (PA5-112426, Thermo) anti-GBP2 (LS-C114752) and anti-TREM2 (AF1729, R&D)<br><br>Secondary Antibodies:Secondary antibodies: goat anti-rabbit Alexa Fluor 488 (Invitrogen, A11008, 1:100), goat anti-mouse Alexa Fluor 488 (Invitrogen, A32723, 1:100), goat anti-rabbit Alexa Fluor 568 (Invitrogen, A11036, 1:100), goat anti-mouse Alexa Fluor 568 Invitrogen, A11031, 1:100), goat anti-mouse HRP conjugated (Invitrogen, 626820, 1:500), goat anti-rabbit HRP conjugated (Invitrogen, 31460, 1:500).
Validation	Antibodies available antibodies were validated for immunoprecipitation, westernblot and immunofluorescences according to their manufacturer.

Animals and other organisms

Policy information about studies involving animals; ARRIVE guidelines recommended for reporting animal research

Laboratory animals	For imaging experiments: Tg-FDD, APP/PS1 or WT (C57/BL6J) mice were sacrificed at 15 months old.<br>For primary cultures: postnatal day 0-3 WT (C57/BL6J) mice were used.

Wild animals	No wild animals were used in this study.
Field-collected samples	No field-collected animals were used in this study.
Ethics oversight	All mice procedures were performed in accordance with the Guide for the Care and Use of Laboratory Animals (National Institutes of Health, Bethesda, MD). Mice were also anesthetized and euthanized according to IUSM Institutional Animal Care and Use Committee-approved procedures.

Note that full information on the approval of the study protocol must also be provided in the manuscript.
